# Advances in Targeted Therapy for Systemic Lupus Erythematosus: Current Treatments and Novel Approaches

**DOI:** 10.3390/ijms26030929

**Published:** 2025-01-23

**Authors:** Kazusa Saegusa, Yumi Tsuchida, Toshihiko Komai, Haruka Tsuchiya, Keishi Fujio

**Affiliations:** Department of Allergy and Rheumatology, Graduate School of Medicine, The University of Tokyo, 7-3-1 Hongo, Bunkyo-ku, Tokyo 113-8655, Japan; saegusa-kazusa086@g.ecc.u-tokyo.ac.jp (K.S.); komait-int@h.u-tokyo.ac.jp (T.K.); tsuchiyah-int@h.u-tokyo.ac.jp (H.T.); fujiok-int@h.u-tokyo.ac.jp (K.F.)

**Keywords:** systemic lupus erythematosus, biologic therapies, targeted therapies, precision medicine

## Abstract

Systemic lupus erythematosus (SLE) is a chronic autoimmune disease with diverse clinical manifestations that can lead to severe organ damage. The complex pathophysiology of SLE makes treatment selection difficult. This review examines the current evidence for biological therapies in SLE, including the anti-B cell activating factor antibody belimumab; the type I interferon receptor antagonist anifrolumab; the novel calcineurin inhibitor voclosporin; and rituximab, which targets CD20 on B cells. We also describe emerging therapies, including novel agents in development and CD19-directed chimeric antigen receptor (CAR) T cell therapy, which has shown promise in early clinical experience. Recent advances in biomarker research, including interferon signatures and transcriptomic profiles, may facilitate patient stratification and treatment selection. This review offers insights into current and future treatment strategies for patients with SLE by analyzing clinical trial results and recent immunological findings.

## 1. Introduction

Systemic lupus erythematosus (SLE) is a chronic autoimmune disease with diverse clinical manifestations and pathophysiological mechanisms. The complex pathophysiology of SLE and heterogeneity among patients make treatment selection difficult.

Immune dysregulation in SLE involves both innate and adaptive immune responses. A loss of immune tolerance leads to the production of autoantibodies against nuclear antigens and other self-antigens. The formation and deposition of immune complexes in various organs trigger inflammation and tissue damage. Key pathogenic features include the excessive production of type I interferon by plasmacytoid dendritic cells, imbalanced T cell responses with impaired regulatory T cell function, B cell hyperactivity driven by elevated levels of B cell activating factor (BAFF), promoting pathogenic autoantibody production, and impaired clearance of apoptotic cells, which provides a source of autoantigens [1].

Even with current standard treatments, including glucocorticoids and immunosuppressants, a recent analysis of the international Definition Of Remission In SLE (DORIS) cohort demonstrated that only 50% of 1652 patients achieved remission or low disease activity [2]. Although glucocorticoids are essential for rapid disease control, their toxicity profile presents a major concern in clinical practice. The short-term adverse effects include an increased risk of serious infections and early mortality [3], while long-term use leads to cumulative organ damage, including osteonecrosis, osteoporosis, and cardiovascular complications [4,5], with damage correlating with glucocorticoid dose and duration [6]. 

The therapeutic approach to SLE has changed significantly over the past decade, shifting from conventional immunosuppression to targeted therapies that aim to reduce both disease activity and treatment-related damage. The currently available approved targeted therapies for SLE include belimumab as the initial biological agent, followed by anifrolumab. For lupus nephritis (LN), the novel calcineurin inhibitor voclosporin has gained approval. Although not approved in many countries, rituximab is widely used in clinical practice, particularly for refractory cases. Recent recommendations from the European League Against Rheumatism (EULAR) [7] and Kidney Disease: Improving Global Outcomes (KDIGO) guidelines on LN [8] have increasingly emphasized the importance of these biological agents and the novel calcineurin inhibitor.

**Figure 1:**
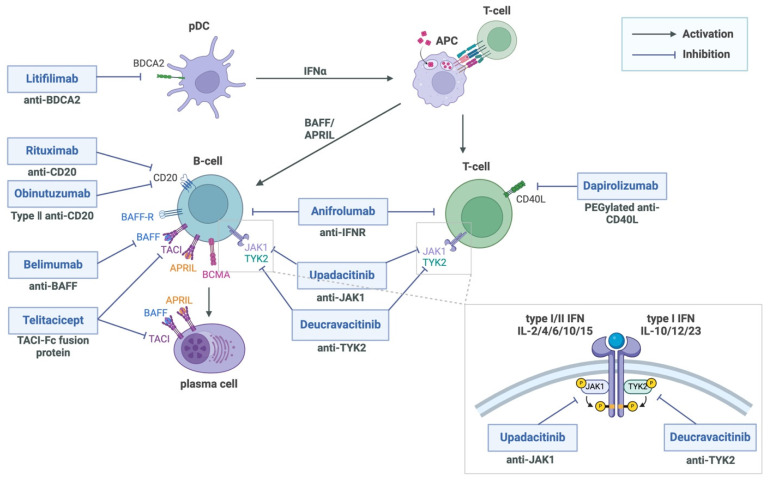
Therapeutic targets in systemic lupus erythematosus. The figure shows molecular targets of current and emerging SLE therapies. Approved therapies include belimumab (targeting BAFF) and anifrolumab (targeting IFNAR). Emerging therapies target various pathways: litifilimab (BDCA2 on pDCs), rituximab and obinutuzumab (CD20 on B cells), telitacicept (BAFF/APRIL), and dapirolizumab (CD40L on T cells). Janus kinase (JAK) inhibitors upadacitinib and deucravacitinib target JAK1 and TYK2, respectively, modulating cytokine signaling (shown in inset). APC, antigen-presenting cells; APRIL, A proliferation-inducing ligand; BAFF, B cell activating factor; BAFF-R, B cell activating factor receptor; BCMA, B cell maturation antigen; BDCA2, blood dendritic cell antigen 2; IFN, interferon; IFNAR, interferon receptor; JAK1, Janus kinase 1; pDC, plasmacytoid dendritic cells; TACI, Transmembrane Activator and Calcium Modulator and Cyclophilin Ligand Interactor; TYK2, tyrosine kinase 2. Figure prepared with BioRender.

Recent clinical data show responses to CD19-directed chimeric antigen receptor (CAR) T cell therapy and T cell engager (TCE) therapy in patients with refractory SLE. Other treatments under investigation include new biological agents, small molecule inhibitors, and cell-based therapies, with a focus on those currently in or with potential for phase III trials. These treatments target specific pathways in SLE pathogenesis, reflecting our current understanding of disease mechanisms (Figure 1).

The availability of multiple therapeutic options with different mechanisms requires careful treatment selection. Patient-specific factors, including disease manifestations, biomarker profiles, and genetic factors, guide treatment decisions. This review examines the clinical evidence for current targeted therapies in SLE, discusses emerging treatments, and considers approaches to treatment selection.

## 2. Current Landscape of Evidence-Based and Novel Therapies in Systemic Lupus Erythematosus

### 2.1. Belimumab

Belimumab is a human monoclonal antibody targeting B cell activating factor/B lymphocyte stimulator (BAFF/BLyS). BAFF is a key regulator in B cell survival, differentiation, and maturation. In SLE, excess BAFF expression contributes to autoreactive B cell survival and autoantibody production. Belimumab binds to soluble BAFF, blocking its interaction with B cell receptors and reducing B cell survival.

Two phase III trials established the clinical efficacy of belimumab. BLISS-52 [9] and BLISS-76 [10] enrolled seropositive patients with active SLE (Safety of Estrogens in Lupus Erythematosus National Assessment–SLE Disease Activity Index (SELENA-SLEDAI) score ≥ 6) but excluded those with severe active LN or central nervous system involvement. Both trials showed higher Systemic Lupus Erythematosus Responder Index (SRI-4 [11]—a composite index requiring a ≥ four-point improvement in clinical Systemic Lupus Erythematosus Disease Activity Index 2000 (SLEDAI-2K)—score, no new severe organ involvement evaluated with the British Isles Lupus Assessment Group (BILAG) index, no worsening in the Physician’s Global Assessment (PGA) by ≥0.3-point response rates at week 52 with belimumab 10 mg/kg compared to placebo (BLISS-52: 58% vs. 44%; BLISS-76: 43.2% vs. 33.5%), and reduced severe flare rates (BLISS-52: 14% vs. 23%; BLISS-76: 20.5% vs. 26.5%). The safety profiles of belimumab, including the rates of serious infections, malignancies, and death, were similar between the belimumab and placebo groups. Subsequent analyses identified high disease activity, positive anti-dsDNA antibodies, and low complement levels as response predictors [12].

The BLISS-LN phase III trial [13] evaluated belimumab for active LN, a condition that had been excluded from the earlier BLISS-52 and BLISS-76 trials. At week 104, belimumab added to standard therapy improved both primary efficacy renal response (43% vs. 32%) and complete renal response (30% vs. 20%). Rates of adverse events were comparable between the two groups across 104 weeks. A post-hoc analysis demonstrated a 55% reduction in renal flares compared with standard-of-care alone [14].

Clinical experience with belimumab, including the 10-year OBSErve cohort [15], shows sustained improvements in disease activity and reduced organ damage. A Cochrane systematic review [16] of six randomized controlled trials further supported belimumab’s efficacy, demonstrating improved disease activity and glucocorticoid-sparing effects compared to placebo, with no significant differences in rates of serious adverse events, serious infections, or mortality between belimumab and placebo.

A multicenter observational study in Italy revealed that the initiation of belimumab in early disease stages was associated with improved clinical outcomes [17]. The key predictors of treatment response included baseline SLEDAI-2K scores ≥ 10, disease duration ≤ 2 years, and minimal organ damage. The analysis demonstrated consistent SRI-4 response rates ranging from 49.2% to 66.7% throughout 48 months of follow-up, accompanied by a marked decrease in disease flares. These findings suggest that patients with limited organ damage and high disease activity will achieve optimal therapeutic benefits from belimumab.

The EULAR recommendations [7] support adding belimumab to standard-of-care in patients who do not respond to hydroxychloroquine or cannot reduce glucocorticoids to acceptable maintenance doses. For active proliferative LN, belimumab can be combined with glucocorticoids and either mycophenolate or low-dose intravenous cyclophosphamide. The KDIGO guideline [8] also includes belimumab with mycophenolate or low-dose intravenous cyclophosphamide among four first-line options, particularly for patients with high flare risk or progressive kidney disease.

### 2.2. Anifrolumab

Anifrolumab is a human monoclonal antibody that targets the type I interferon receptor 1 (IFNAR1). By blocking IFNAR1, anifrolumab inhibits signaling of all type I interferons, including IFNα, IFNβ, and IFNω. Type I interferons play a central role in the pathogenesis of SLE [18]. A significant proportion of patients with SLE show elevated interferon-regulated gene expression, characterized by the interferon gene signature (IGS).

The efficacy of anifrolumab was investigated in two phase III trials [19]. TULIP-1 [20] did not meet its primary endpoint using the SRI-4, possibly due to the limitations of the SRI-4 in capturing subtle improvements in disease activity. In contrast, TULIP-2 [21] demonstrated the superiority of anifrolumab over placebo using the British Isles Lupus Assessment Group-based Composite Lupus Assessment (BICLA [22], a composite index based on the BILAG-2004 (an updated version of BILAG, incorporating nine organ systems and refined scoring to improve sensitivity), requiring improvement in all BILAG A or B scores at baseline, no new BILAG A or B scores, stable SLEDAI-2K, and no worsening in the PGA (defined as an increase of <0.3 points on a three-point scale) at week 52 (47.8% vs. 31.5%). Both trials showed significant improvements in skin disease and reduced oral glucocorticoid use. In TULIP-2, patients with high IGS showed a higher treatment response rate (48.0% vs. 30.7%) compared with those with low signatures (46.7% vs. 35.5%). Herpes zoster (HZ) occurred more frequently with anifrolumab than with placebo (7.2% vs. 1.1%). A post-hoc analysis of the pooled TULIP trials demonstrated that anifrolumab-treated patients achieved the Lupus Low Disease Activity State (LLDAS) more frequently than placebo (30.0% vs. 19.6%) at week 52 [23]. 

The MUSE phase IIb study showed significant improvements in rash (SLEDAI-2K resolution: 44.3% vs. 14.8%) and arthritis (SLEDAI-2K improvement: 56.7% vs. 42.4%) compared to placebo [24], with these benefits maintained in its 3-year open-label extension study [25]. 

The TULIP 3-year extension study also showed sustained improvement in disease activity scores and lower cumulative glucocorticoid use [26]. Anifrolumab demonstrated an overall acceptable safety profile, with an increased risk of HZ during the initial treatment phase that decreases over time. Most HZ cases were manageable with antiviral therapy, and the rates of serious infections (3.7 vs. 3.6 per 100 patient-years) and serious adverse events (8.5 vs. 11.2 per 100 patient-years) were comparable to those in the placebo group. Due to the timing of the recombinant HZ vaccine approval during the study period, few patients received vaccination. The TULIP-LN phase II trial [27] provided additional evidence for anifrolumab’s potential efficacy in LN, with the intensified regimen group showing higher complete renal response rates compared to the placebo group (45.5% vs. 31.1%), with response criteria including the normalization of proteinuria and the stabilization of renal function. The safety profiles were almost similar in both groups, though herpes zoster occurred more frequently in the anifrolumab group (16.7% vs. 8.2%).

The recent EULAR recommendations [7] position anifrolumab as an option for first-line therapy in non-renal SLE, alongside belimumab, without requiring prior failure of conventional immunosuppressants, especially for active skin disease. While the safety profile of anifrolumab is acceptable, careful monitoring of HZ is needed. There is limited evidence for active neuropsychiatric SLE, as such cases were excluded from trials.

### 2.3. Rituximab

Rituximab is a chimeric monoclonal antibody targeting CD20 on B cells, which spares plasma cells and early B cell precursors. While not approved by the US Food and Drug Administration or the European Medicines Agency, clinical experience supports its use in specific clinical settings, particularly in patients with severe or refractory cases.

Two randomized controlled trials, the EXPLORER phase II/III trial [28] for non-renal SLE and the LUNAR phase III trial [29] for LN, did not meet their primary endpoints. The EXPLORER trial assessed rituximab in moderate-to-severe non-renal SLE but found no difference in clinical response between rituximab and placebo at 52 weeks. In the LUNAR trial, which evaluated rituximab with mycophenolate and corticosteroids in class III/IV LN, overall renal response rates were not significantly different (56.9% vs. 45.8%). However, rituximab-treated patients showed greater reductions in anti-dsDNA antibodies, and none required cyclophosphamide rescue therapy, compared to eight patients in the placebo group.

Registry data support the clinical value of rituximab in specific situations. The British Isles Lupus Assessment Group Biologics Register reported reduced disease activity scores and lower glucocorticoid doses in rituximab-treated patients. Serious infections were reported in 10% of patients, with higher frequency in the first 3 months post-rituximab [30]. The French Autoimmunity and Rituximab registry, with 136 patients with SLE, documented clinical responses in severe manifestations, including LN, neuropsychiatric involvement, and hematologic abnormalities. Severe infections occurred in 9% of patients (6.6/100 patient-years), mainly within 3 months after the last infusion [31]. Although the safety data from registry studies was acceptable, infection risk monitoring is important in the first 3 months after rituximab administration.

For severe autoimmune thrombocytopenia in SLE, rituximab is effective as part of acute treatment along with high-dose glucocorticoids (including intravenous methylprednisolone pulses) and/or intravenous immunoglobulin, and/or high-dose intravenous cyclophosphamide [7]. A multicenter study of 71 adults with SLE-associated immune cytopenia showed its efficacy, with a 91% response rate in patients with immune thrombocytopenia. Severe infections were reported in 4.2% of patients treated with rituximab, with no fatal outcomes [32]. Rituximab can also be considered for maintenance therapy, with alternatives such as azathioprine, mycophenolate, or cyclosporine.

Clinical trials have examined rituximab with belimumab. The CALIBRATE phase II trial [33] tested rituximab induction followed by belimumab in patients with refractory LN, finding similar safety profiles between groups. The BEAT-LUPUS phase II trial [34] compared rituximab followed by belimumab versus rituximab followed by placebo, with higher major clinical response rates at week 52 in the belimumab group (48% vs. 35%). The trial identified baseline serum IgA2 anti-dsDNA antibodies as a potential response biomarker, showing a 48% higher response rate in patients with elevated baseline levels. The BLISS-BELIEVE phase III trial [35] compared subcutaneous belimumab with rituximab (BEL/RTX) versus belimumab with placebo (BEL/PBO). While BEL/RTX did not increase disease control rates (SLEDAI-2K ≤ 2, without immunosuppressants, prednisone ≤ 5 mg/day) at week 52 (19.4% vs. 16.7%), these patients maintained disease control longer and showed greater reductions in anti-dsDNA antibodies and B cell numbers. The combination of rituximab with belimumab did not increase adverse events compared to rituximab alone, as demonstrated in both the CALIBRATE and BLISS-BELIEVE trials [33,35]. These results point to the potential of B cell-targeted combination therapy with biomarker guidance for patient selection.

### 2.4. Voclosporin

Voclosporin is a novel calcineurin inhibitor structurally modified from cyclosporine. Compared to conventional calcineurin inhibitors, it demonstrates higher potency in inhibiting T cell activation and IL-2 transcription, with more predictability, eliminating the need for therapeutic drug monitoring.

The AURORA-1 phase III trial [36] demonstrated its efficacy in LN as an add-on therapy to mycophenolate and low-dose steroids. At week 52, complete renal response rates were significantly higher with voclosporin versus placebo (41% vs. 23%). Serious adverse events occurred in 21% of patients in both groups, with pneumonia being the most frequent serious infection (4% in both groups). However, the trial did not assess renal biopsy activity and chronicity indices or evaluate responses between new-onset and relapsed LN.

The AURORA-2 extension study [37] provided evidence for long-term safety and efficacy over 3 years. Among 216 patients, 86.1% completed the study with no unexpected safety concerns reported. While glomerular filtration rate (GFR) decreases and hypertension were more frequent with voclosporin (10.3% vs. 5.0% and 8.6% vs. 7.0%, respectively), the mean estimated GFR (eGFR) remained stable and within the normal range. The eGFR slope over 2 years was −0.2 mL/min/1.73 m^2^ with voclosporin versus −5.4 mL/min/1.73 m^2^ with placebo. Complete renal response rates increased to 50.9% with voclosporin versus 39.0% with control at 3 years. Notably, the AURORA trials demonstrated efficacy with lower glucocorticoid doses (20–25 mg/day prednisone, rapidly tapered to 5 mg by 12 weeks) than traditional regimens. While voclosporin was studied across LN classes, its efficacy in pure class V LN requires further investigation due to limited patient numbers in clinical trials. A limitation of AURORA-2 was that the voclosporin group had higher renal response rates at study entry, potentially affecting the interpretation of long-term outcomes.

The EULAR recommendations [7] note that voclosporin provides a rapid reduction in proteinuria, which may be beneficial in patients with high baseline proteinuria in the nephrotic range. The KDIGO guideline [8] states that voclosporin should be used cautiously in patients with impaired kidney function (eGFR < 45 mL/min/1.73 m^2^) or with widespread sclerotic changes on kidney biopsy. It also states that treatment continuation for up to 3 years appears safe, as supported by stable GFR levels in long-term studies.

## 3. Emerging Therapies

### 3.1. Novel Biological Agents in Development

Ongoing research has yielded new therapeutic approaches for SLE beyond current standard treatments. This section examines selected agents showing particular promise in phase II and III studies (Table 1).

- Obinutuzumab

Obinutuzumab, a type II anti-CD20 monoclonal antibody, differs from rituximab in its CD20 binding properties and mechanisms of B cell depletion. In vitro studies demonstrate superior B cell cytotoxicity compared to rituximab through enhanced Fc gamma receptor-mediated effects and direct cell death, maintaining surface localization after CD20 binding [38].

The NOBILITY phase II trial in LN showed that the addition of obinutuzumab to standard therapy achieved higher complete renal response rates compared to placebo at week 52 (35% vs. 23%) and week 104 (41% vs. 23%), with good tolerability except for increased non-serious infusion reactions [39]. Furthermore, in patients with renal or non-renal SLE with secondary non-response to rituximab, obinutuzumab improved disease activity scores and achieved B cell depletion, suggesting its potential role as an alternative therapy in refractory cases [40]. These results supported the initiation of an ongoing phase III trial.

- Dapirolizumab Pegol

Dapirolizumab pegol is a PEGylated anti-CD40L antibody fragment that blocks CD40-CD40L interactions, which are key pathways in T cell-dependent B cell responses and in autoimmune inflammation in SLE. Early attempts to target this pathway with conventional anti-CD40L antibodies showed promise but were halted due to thromboembolic events [41]. Dapirolizumab pegol was designed without an Fc domain to avoid platelet activation while maintaining therapeutic efficacy.

In a phase II trial of patients with moderately to severely active SLE [42], intravenous dapirolizumab pegol (6/24/45 mg/kg) showed clinical and immunological improvements across all doses compared to placebo at 24 weeks, though a clear dose–response relationship was not established. In addition, dapirolizumab pegol demonstrated an acceptable safety profile with no increased thrombotic risk.

The phase III PHOENYCS GO trial [43] demonstrated superior efficacy of dapirolizumab pegol (24 mg/kg) compared to placebo in moderate-to-severe SLE patients. The primary endpoint showed significantly higher BICLA response rates in the treatment group (49.5% vs. 34.6%), along with improved SRI-4 responses (60.1% vs. 41.1%) and reduced severe flares (11.6% vs. 23.4%). While treatment-related adverse events were more frequent with dapirolizumab pegol, serious adverse events were less common, suggesting an acceptable safety profile.

The PEGylated structure of dapirolizumab pegol may limit placental transfer, suggesting potential use during pregnancy pending further safety data.

- Telitacicept

As discussed in the belimumab section, B cells play a central role in SLE pathogenesis through autoantibody production and immune dysregulation. Telitacicept is a fusion protein that combines the extracellular domain of the transmembrane activator and calcium modulator and cyclophilin ligand interactor (TACI) receptor with the Fc portion of human IgG1, targeting two key B cell survival factors: BAFF/BlyS and A proliferation-inducing ligand (APRIL). They promote B cell differentiation, maturation, and survival through binding to cell surface receptors, including TACI. Unlike belimumab, which selectively inhibits BlyS, telitacicept blocks both the BlyS and APRIL signaling pathways, potentially offering the complete suppression of B cell-mediated autoimmunity. This dual inhibition may provide more comprehensive control of aberrant B cell responses in SLE.

In a phase IIb randomized controlled trial of 249 patients with active SLE in China [44], subcutaneous telitacicept (80 mg, 160 mg, or 240 mg weekly) demonstrated significantly higher SRI-4 response rates at week 48 across all treatment groups compared to placebo (71.0–75.8% vs. 33.9%). The 240 mg dose showed additional benefits, including glucocorticoid dose reduction. The safety profile was comparable to placebo, suggesting good tolerability. Further investigations are needed to confirm its efficacy and safety, including larger trials in more diverse populations. Phase III clinical trials are set to be conducted at multiple sites across 15 countries [45].

- Litifilimab

Litifilimab is a humanized IgG1 monoclonal antibody designed to target blood dendritic cell antigen 2 (BDCA2), which is uniquely expressed on plasmacytoid dendritic cells. By binding to BDCA2, litifilimab suppresses the production of type I interferons, cytokines, and chemokines—key mediators in SLE pathogenesis. This mechanism provides a novel approach to modulating the interferon pathway in SLE, distinct from direct cytokine or receptor blockade.

In a phase II trial (LILAC) [46], subcutaneous litifilimab (450 mg) demonstrated superiority over placebo in reducing active joint count at 24 weeks in patients with SLE with arthritis and active skin disease; however, the results of most secondary endpoints were not consistent with that of the primary endpoint. The safety profile included cases of herpes zoster and herpes keratitis, warranting further evaluation. Phase III trials are currently in progress to confirm the efficacy and safety of litifilimab.

- Upadacitinib

Upadacitinib is a selective Janus kinase 1 (JAK1) inhibitor that blocks signaling through multiple cytokine pathways involved in SLE pathogenesis, including type I and II interferons and the interleukins IL-2, IL-4, IL-6, IL-10, and IL-15. 

In the phase II SLEek trial [47], more patients who received upadacitinib 30 mg once daily achieved the SRI-4 response compared with placebo at week 24 (54.8% vs. 37.3%). It also demonstrated improvements in BICLA response and LLDAS and fewer disease flares. The safety profile was consistent with that observed in other approved indications, such as rheumatoid arthritis and psoriatic arthritis. Further investigation of upadacitinib for moderate to severe SLE in phase III trials is under way. It was also evaluated in combination with the BTK inhibitor elsubrutinib (ABBV-599), showing comparable efficacy to upadacitinib monotherapy but requiring additional studies to determine the optimal therapeutic approach.

- Deucravacitinib

Deucravacitinib is an orally administered, selective, allosteric inhibitor of tyrosine kinase 2 (TYK2) that targets its regulatory domain and inhibits signaling pathways mediated by type I interferons as well as the interleukins IL-10, IL-12, and IL-23, which are key cytokines in SLE pathogenesis.

In a phase II trial involving 363 patients with active SLE [48], the SRI-4 response rate at week 32 was significantly higher in patients receiving deucravacitinib 3 mg twice daily compared to placebo (58% vs. 34%). The safety profile was generally acceptable, though increased rates of infections and cutaneous events were noted. Further study is needed, including the ongoing Phase III trial [49], to confirm its efficacy and safety.

- Others

Low-dose IL-2 therapy has gained attention as a potential treatment for SLE based on its ability to expand regulatory T cells (Tregs) without activating effector T cells. While the initial results at 12 weeks were not statistically significant in a controlled trial, the follow-up at 24 weeks revealed superior outcomes compared to placebo in both overall response (66% vs. 37%) and nephritis remission (53.85% vs. 16.67%) [50]. Further studies are underway in China [51,52].

Ustekinumab, targeting IL-12/23 p40, demonstrated efficacy in SLE in a phase II trial with higher SRI-4 response rates compared to placebo (62% vs. 33%) [53] but failed to meet its primary endpoint in phase III testing (44% vs. 56% for placebo), resulting in trial discontinuation [54].

Despite encouraging case reports of secukinumab in the treatment of SLE [55,56], clinical trials of this IL-17A inhibitor were unsuccessful [57,58,59]. Cases of drug-induced lupus after treatment for ankylosing spondylitis and psoriasis with IL-17A inhibitors have also been reported [60,61], highlighting the complex role of IL-17 in lupus pathogenesis.

**Table 1:** Clinical trials of novel biological agents in systemic lupus erythematosus.

Agent	Mechanism of Action	Trial Phase	Patient Population	Primary Endpoint (Treatment vs. Placebo)	Key Secondary Outcome
Obinutuzumab [39]	Type II anti-CD20 mAb	II	Active/chronic LN	CRR at week 52: 35% vs. 23%	CRR at week 104: 41% vs. 23%
Dapirolizumab pegol [42]	PEGylated anti-CD40L	III	Moderate to severely active SLE, stable LN	Dose–response relationship of BICLA response rates at week 24: none	BICLA response rate: 48.8–54.5% vs. 37.2%
Telitacicept [44]	TACI-Fc fusion protein (BlyS/APRIL inhibitor)	IIb	Active SLE	SRI-4 response rate at week 48: 71.0–75.8% vs. 33.9%	GC dose reduction with 240 mg dose
Litifilimab [46]	Anti-BDCA2	II	SLE (SLEDAI-2K ≥ 4)	Total number of active joints at week 24: 19.0 ± 8.4 vs. 21.6 ± 8.5	Most secondary endpoints not met
Upadacitinib [47]	JAK1 inhibitor	II	Moderate to severely active SLE	SRI-4 response rate and GC dose ≤ 10 mg QD at week 24: 54.8% vs. 37.3%	SRI-4, BICLA, LLDAS response rate at week 48: 45.2% vs. 32.0%, 53.2% vs. 25.3%, 50.0% vs. 24.0%. Overall flares at week 24: 1.9 vs. 2.8.
Deucravatinib [48]	TYK2 inhibitor	II	Active SLE	SRI-4 response rate at week 32: 58% vs. 34%	BICLA, CLASI-50, LLDAS response rates, active joint count at week 48: 57.1% vs. 34.4%, 47.3% vs. 25.6%, 36.6% vs. 13.3%, −8.9 vs. −7.6.

Abbreviations: APRIL, A proliferation-inducing ligand; BDCA2, blood dendritic cell antigen 2; BICLA, British Isles Lupus Assessment Group-based Composite Lupus Assessment; BlyS, B cell activating factor; CLASI, Cutaneous Lupus Erythematosus Disease Area and Severity Index; CRR, complete renal response; GC, glucocorticoids; JAK1, Janus kinase 1; LLDAS, Lupus Low Disease Activity State; LN, lupus nephritis; SLE, systemic lupus erythematosus; SLEDAI-2K, Systemic Lupus Erythematosus Disease Activity Index 2000; SRI-4, SLE Responder Index 4; TYK2, tyrosine kinase 2.

### 3.2. CAR T Cell Therapy and T Cell Engager Therapy

- CAR T Cell Therapy

CD19-directed chimeric antigen receptor (CAR) T cell therapy, originally developed for B cell malignancies, provides a novel approach for severe SLE. B cells are central to SLE pathogenesis; however, conventional B cell-depleting therapies such as rituximab have shown limited efficacy in clinical trials. This is possibly due to the autoreactive B cells in lymphatic organs and inflammatory tissues and the CD20-negative plasma affecting autoantibody production [62]. CAR T cells are engineered to express receptors targeting CD19 on B cells, leading to profound B cell depletion. Although life-threatening adverse effects, such as high-grade cytokine release syndrome (CRS), immune effector cell-associated neurotoxicity syndrome (ICANS), and immune effector cell-associated hemophagocytic lymphohistiocytosis-like syndrome, have been reported in experiences with malignancies, this approach remains promising. It offers the potential to eliminate disease-driving autoantibodies and promote immune system restoration, particularly in refractory patients [63].

Initial clinical experience with CAR T cell therapy in SLE has yielded promising results. The first reported case was a 20-year-old woman with severe, treatment-refractory SLE who achieved complete clinical and serological remission following CAR T cell infusion [64]. This report was followed by a case series of five patients with refractory SLE, all of whom achieved DORIS remission within 3 months of treatment [65]. A subsequent cohort study of 15 patients with autoimmune diseases, including 8 with SLE, has expanded these observations [66]. Clinical remission continued after B cell reconstitution at approximately 110 days. These reconstituted B cells show a naïve phenotype with non–class-switched B cell receptors, suggesting restoration of B cell tolerance.

CAR T cell therapy safety profiles appear more favorable in SLE than in B cell malignancies. In patients with cancer, life-threatening adverse effects, such as high-grade CRS and immune effector cell-associated hematotoxicity, rarely occur in patients with SLE. This possibly reflects the lower burden of target cells in SLE than in B cell malignancies [63].

Dual-targeting strategies are also being explored. The B cell maturation antigen (BCMA)-CD19 compound CAR approach targets both B cells and plasma cells. Phase I trial data showed patients testing negative for all disease-associated autoantibodies and sustaining medication-free remission for up to 46 months [67].

Several questions remain concerning appropriate patient selection, long-term safety, and durability of response. The treatment requires specialized centers capable of T cell processing and managing potential complications. Further studies, including controlled clinical trials, are needed to establish the role of CAR T cell therapy in severe SLE. Further understanding of post-reconstitution B cell tolerance may guide future therapeutic strategies.

- T Cell Engager Therapy

Another emerging approach involves T cell engagers (TCEs). This approach uses bispecific antibodies to simultaneously bind CD3 on T cells and specific antigens on target cells, such as B cell maturation antigen (BCMA) or CD19 on B cells and plasma cells, thereby activating T cells to mediate the targeted destruction of pathogenic cells in autoimmune diseases. Initially developed for the treatment of hematological malignancies, TCEs offer several practical advantages over CAR T therapy: no requirement for complex manufacturing processes or lymphodepleting chemotherapy, lower costs, and easier dose modification based on clinical response. While both TCEs and CAR T therapy share common adverse events including CRS, ICANS, infections, and hematological toxicities, preliminary data suggest better tolerability of TCEs in autoimmune diseases than in cancer treatment [68,69].

Teclistamab, which targets CD3 and BCMA and is used in relapsed or refractory multiple myeloma [70], has demonstrated promise in treating refractory autoimmune diseases, including systemic sclerosis, Sjögren’s syndrome, and rheumatoid arthritis (RA) [68]. In a case report of a young woman with refractory SLE treated with teclistamab [69], the patient experienced complete remission through effective B cell and plasma cell depletion. The drug showed a favorable safety and tolerability profile, with manageable adverse events, including low-grade cytokine release syndrome. Based on these encouraging results with BCMA-targeting therapy, other agents in this class warrant investigation. Among them, elranatamab, another CD3-BCMA TCE approved for multiple myeloma, offers potential advantages, including a longer half-life (22 days vs. 15 days) and fixed dosing rather than weight-based administration [71]. 

Blinatumomab, targeting CD3 and CD19, achieved synovial B cell depletion in two-thirds of RA patients resistant to conventional therapy, demonstrating superior tissue penetration compared to rituximab [72]. In this study, treatment led to significant clinical improvement and reduction in autoantibody levels. Both rituximab and CD19-targeted therapies share a common limitation: CD19-negative long-lived plasma cells remain unaffected, leading to persistent autoantibody production in some patients. This limitation highlights the potential therapeutic advantage of CD3-BCMA TCEs in certain cases, as they can target both B cells and plasma cells expressing BCMA. A trial is currently underway for a new CD19/CD3/human serum albumin-targeting TCE in SLE, designed to direct T cells to eliminate B cells, including those with diminished or undetectable CD19 expression, as determined by immunohistochemistry [73,74]. 

Further studies are needed to establish the efficacy and safety profile of this approach. Key future challenges include determining suitable patients for TCE therapy and establishing appropriate dosing protocols in autoimmune conditions.

## 4. Future Perspectives: Precision Medicine in Systemic Lupus Erythematosus

Despite the recent advances in treatment discussed above, SLE remains challenging to manage due to its heterogeneous clinical presentation and unpredictable disease course. Current SLE classification relies primarily on clinical manifestations and serological findings, often failing to capture the underlying molecular heterogeneity driving disease activity and organ damage. The concept of precision medicine offers the potential to overcome this limitation in SLE treatment by tailoring therapies to individual patient characteristics and disease mechanisms.

- Genetic Factors in SLE Pathogenesis and Patient Stratification

Genetic factors, as well as epigenetic factors, including DNA methylation, histone modifications, and microRNAs, also contribute to this diversity [75]. Genome-wide association studies (GWAS) have identified over 100 SLE risk loci, many of which are involved in immune regulation, interferon signaling, and B cell function [1,75]. Variants in interferon pathway genes, including *IRF5* and *STAT4,* are associated with altered responses to type I interferons. Polymorphisms in genes encoding B cell signaling molecules, such as *BLK* and *BANK1*, have been associated with altered B cell activation and autoantibody production [76,77]. Additionally, defects in genes involved in nucleic acid degradation and sensing, including *DNASE1* and *DNASE1L3*, contribute to disease development in some patients [1,78,79]. Understanding these genetic variations could help identify patients at risk for disease development and guide therapeutic monitoring.

The identification of organ-specific genetic associations has particular relevance for treatment selection. Variants in platelet-derived growth factor receptor alpha (*PDGFRA*) and *HAS2* have been linked to lupus nephritis development [75,80,81]. *ITGAM* variants show an association with cutaneous, joint, and neuropsychiatric manifestations in addition to renal impairment [75,82,83,84]. These genetic markers may help identify patients most likely to benefit from specific targeted therapies.

Advancements in transcriptomics provide further insights into genetic factors in SLE. In a study of 162 SLE patients and 99 controls, single-cell RNA sequencing of peripheral blood mononuclear cells showed that monocytes had the most prominent type I IGS among eight cell types. Monocyte IGS inversely correlated with naive CD4^+^ T cell counts, and cell-type-specific expression patterns could stratify patients into two molecular subtypes [85]. These genetic associations uncover important aspects of the molecular pathways driving SLE pathogenesis.

Epigenetic modifications also affect gene expression and immune cell function in SLE, with DNA hypomethylation and histone changes modulating CD4^+^ T cell activity [75,86]. MicroRNAs regulate inflammatory pathways and cytokine production, suggesting their potential as disease biomarkers [75,87]. These findings could guide personalized treatment approaches.

- Biomarker-Based Patient Stratification

The heterogeneity of SLE reflects diverse underlying immunological conditions involving various cell types, including T cells, B cells, neutrophils, dendritic cells, and platelets, as well as the dysregulation of cytokines, complement pathways, and other immune mediators [68]. Identifying and validating biomarkers that reflect these diverse pathophysiological processes is needed to stratify patients accurately. 

Several potential biomarkers are currently under investigation. The IGS reflects the increased expression of interferon-stimulated genes, a common feature in SLE that is often associated with disease activity and specific organ manifestations [69]. Circulating proteins, such as CXCL10, galectin-9, and sialic acid-binding Ig-like lectin 1 (Siglec-1), are promising surrogate biomarkers of type I interferon for identifying patients at risk of flares or those likely to benefit from interferon-targeted therapies. CXCL10 is a chemokine involved in inflammation and autoimmune diseases [75], with elevated levels in patients with SLE [88], making it a potential marker for disease activity and flares [89], especially in LN and neuropsychiatric symptoms [88,90,91]. Galectin-9 correlates with disease activity and damage and is a potential marker for neuropsychiatric SLE [92]. Siglec-1, a myeloid cell-surface protein, correlates with IGS and renal complications, though not with disease activity [93].

Serum levels of BAFF and APRIL, cytokines that promote B cell survival and differentiation, are also being investigated. As discussed in the belimumab and the telitacicept sections, elevated levels are associated with SLE disease activity and may predict response to therapies targeting these pathways [75,94].

Urinary biomarkers can reflect kidney involvement in SLE. Monocyte chemoattractant protein-1 is elevated in active LN [95]. TNF-like weak inducer of apoptosis is associated with renal inflammation and may predict treatment response [96]. Neutrophil gelatinase-associated lipocalin is predictive of flares in LN [97].

Enzymes associated with lysophospholipid production are also under investigation [98].

Advanced technologies, such as transcriptomic analyses and mass cytometry, enable more detailed immune profiling, potentially revealing novel biomarkers that reflect specific disease endotypes [75]. A recent study from Japan identified distinct transcriptomic signatures associated with disease establishment and exacerbation, offering potential biomarkers for predicting disease course and treatment response [99]. For example, neutrophil-related signatures have been associated with renal involvement [99]. Transcriptomic analyses have provided a comprehensive view of immune-cell–specific gene regulation in SLE, revealing distinct pathways linked to disease phases. Single-cell RNA sequencing has revealed disease activity-dependent transcriptomic changes [85]. Machine learning may help transcriptomic analyses and contribute to patient stratification [100].

- Predicting Treatment Response

Predicting which patients are most likely to benefit from a given therapy is a key goal of precision medicine, which is particularly crucial in SLE, where treatment responses can vary widely. For example, patients with high IGS may be more likely to respond to anifrolumab [95]. 

Further refinement of treatment response prediction may come from understanding the dynamic interplay between genetic variants, gene expression, and environmental factors (context-dependent expression quantitative trait loci or eQTLs) [101]. For example, context-dependent eQTLs affecting genes such as NXF1 in monocytes could influence responses to therapies targeting Toll-like receptor 7 pathways. Another study demonstrated the potential of disease-activity signatures for predicting treatment response, showing that good responders to belimumab exhibited transcriptomic changes that counteracted these signatures [99]. This suggests that monitoring such signatures could help assess treatment efficacy and guide therapeutic decisions. 

Realizing precision medicine in SLE faces several challenges. Further research is needed to standardize these biomarker assays and validate them across diverse populations before widespread adoption.

## 5. Conclusions

Recent advances in targeted therapies have expanded treatment options for patients with SLE. Clinical evidence supports the efficacy of B cell targeted therapy with belimumab, particularly in patients with high disease activity and positive serological markers. The type I interferon receptor antagonist anifrolumab has shown benefits, especially in patients with active skin disease and high IGS. For LN, voclosporin provides rapid proteinuria reduction with predictable pharmacokinetics. While not approved in many countries, rituximab remains valuable for specific manifestations, including severe cytopenia and refractory disease.

Several promising agents are under investigation, including obinutuzumab, dapirolizumab pegol, and telitacicept, each targeting distinct pathways in SLE pathogenesis. Novel approaches such as dual-targeting strategies and small molecule inhibitors may further expand therapeutic options. Early clinical experiences with CAR T cell therapy and TCE therapy suggest their potential in severe, treatment-resistant cases. 

The shift toward precision medicine in SLE treatment requires improved patient stratification based on molecular and clinical characteristics. Research into biomarkers, including interferon signatures and transcriptomic profiles, may help identify patients most likely to benefit from specific therapies. Challenges remain in standardizing these biomarker assays and validating them across diverse populations.

Additional research is required to identify reliable predictors of treatment response, understand treatment resistance mechanisms, and develop strategies for treatment sequencing or combination. A growing understanding of SLE pathogenesis may contribute to realizing tailored therapies to achieve sustained remission while minimizing organ damage.

## Abbreviations

APC	antigen-presenting cells
APRIL	A proliferation-inducing ligand
BAFF/BlyS	B cell activating factor/B lymphocyte stimulator
BAFF-R	B cell activating factor receptor
BCMA	B cell maturation antigen
BDCA2	blood dendritic cell antigen 2
BEL/PBO	belimumab with placebo
BEL/RTX	belimumab with rituximab
BICLA	British Isles Lupus Assessment Group-based Composite Lupus Assessment
BILAG	British Isles Lupus Assessment Group (index)
CAR	chimeric antigen receptor
CD	cluster of differentiation
CD40L	cluster of differentiation 40 ligand
CRS	cytokine release syndrome
CXCL10	cysteine-X-cysteine motif chemokine ligand 10
DORIS	Definition Of Remission In SLE
eGFR	estimated glomerular filtration rate
eQTLs	expression quantitative trait loci
EULAR	European League Against Rheumatism
GFR	glomerular filtration rate
GWAS	genome-wide association studies
HZ	herpes zoster
ICANS	immune effector cell-associated neurotoxicity syndrome
IFN	interferon
IFNAR	interferon receptor
IFNAR1	interferon receptor 1
IGS	interferon gene signature
Ig	immunoglobulin
IL	interleukin
JAK	Janus kinase
JAK1	Janus kinase 1
KDIGO	Kidney Disease: Improving Global Outcomes
LLDAS	Lupus Low Disease Activity State
LN	lupus nephritis
pDC	plasmacytoid dendritic cells
PDGFRA	platelet-derived growth factor receptor alpha
PGA	Physician’s Global Assessment
RA	rheumatoid arthritis
SELENA-SLEDAI	Safety of Estrogens in Lupus Erythematosus National Assessment–Systemic Lupus Erythematosus Disease Activity Index
SLE	systemic lupus erythematosus
SLEDAI-2K	Systemic Lupus Erythematosus Disease Activity Index 2000
SRI-4	Systemic Lupus Erythematosus Responder Index
TACI	transmembrane activator, calcium modulator, and cyclophilin ligand interactor
TCE	T cell engager
Treg	regulatory T cell
TYK2	Tyrosine kinase
